# Spectrum of constitutional chromosomal disorders: A five-year retrospective analysis from a tertiary care center in Karachi

**DOI:** 10.12669/pjms.42.(ICON26).15689

**Published:** 2026-04

**Authors:** Saba Aman, Mustajab Mujtaba, Maliha Naz, Neelum Mansoor

**Affiliations:** 1 Dr. Saba Aman, FCPS. Fellow, Molecular Pathology and Cytogenetics, Indus Hospital and Health Network, Karachi, Pakistan; 2 Dr. Mustajab Mujtaba, FCPS. Consultant Cardiologist, Department of Cardiology, Dr. Ziauddin Hospital, Karachi, Pakistan; 3 Mrs. Maliha Naz, MPhil. Medical Scientist II, Department of Cytogenetics, Indus Hospital and Health Network, Karachi, Pakistan; 4 Dr. Neelum Mansoor, FCPS. Section Head, Department of Cytogenetics, Indus Hospital and Health Network, Karachi, Pakistan

**Keywords:** Constitutional chromosomal abnormalities, Trisomy 21, Constitutional karyotype

## Abstract

**Objective:**

Cytogenetic analysis plays a crucial role in the assessment of genetic disorders. Our study aimed to provide an overview of the prevalence of constitutional chromosomal abnormalities, enabling the recognition of common presentations and frequencies, which would assist us in identifying the disease burden and establishing better diagnostic services for cytogenetic studies.

**Methodology:**

This is a retrospective study conducted at the department of Cytogenetics, Clinical Laboratories of Indus Hospital and Health Network, Karachi, from January 2020 to May 2025. All cases, irrespective of age and gender, were included in the study. Conventional karyotyping is performed on peripheral blood using G-banding. Patients’ demographics and clinical parameters were retrieved from electronic medical records of the hospital after IRB approval (IHHN-IRB NUMBER: IHHN_IRB_023_05_003).

**Results:**

Total 348 cases were received for cytogenetic analysis with suspected constitutional disorder. Median age of the patients was 10 months(one day-55years) with male to female ratio of 0.7. The most common indication was suspected Down syndrome (DS), with suspected Turner syndrome and ambiguous genitalia being among other indications. The most commonly diagnosed disorder was also Down syndrome.

**Conclusion:**

Constitutional chromosomal disorders were found to be relatively frequent in our population, with numerical abnormalities occurring more often than structural abnormalities. Trisomy 21 is the most frequent disorder found in our cohort. Overall findings highlight the diverse spectrum of constitutional chromosomal disorders in Pakistan and underscore the need to strengthen cytogenetic diagnostic services to improve early recognition and patient care.

## INTRODUCTION

A rearrangement in the structure or number of chromosomes that deviates from the normal karyotype in a cell is referred to as a chromosomal abnormality.^1^ Chromosomal abnormalities can be constitutional or acquired. Acquired changes manifest within cells anywhere or anytime during an individual’s lifetime, mostly observed in tumor cells, whereas constitutional abnormality is present from conception in all or most cells and can be inherited or occur spontaneously.^2^ There can be numerous effects of constitutional chromosomal abnormalities on human life, which can be increased pregnancy losses, infertility, congenital abnormalities, intellectual disabilities, etc. The prevalence of chromosomal rearrangements is 2–8% in couples with repeated pregnancy losses as compared to 0.55% of the general population.^3^ They impact at least 7.5% of all conceptions, and most of these anomalies end up in spontaneous abortions, reaching a frequency of 0.6% at live births.^4^

Cytogenetic analysis plays a crucial role in the assessment of genetic disorders, including birth defects, intellectual disability, developmental delay, and cancers.^5^-^7^ With more and more data accumulating for conditions causing a wide spectrum of different behavioral abnormalities in children being diagnosed, it has been found that various microdeletion syndromes lie at the back of such disorders, which can be diagnosed with high-resolution cytogenetic analysis. Even though major diagnostic efforts are being put in for children with respect to conditions like autism spectrum disorders, it is still unknown how many adults with behavioral abnormalities carry these microdeletions.

The spectrum of constitutional disorder in our region is not explored to the extent that it should have been. Increasing cases of infertility must drive our instincts to assess for the presence of constitutional disorders. Our study aimed to provide an overview of the prevalence of constitutional chromosomal abnormalities, enabling the recognition of common presentations and frequencies, which would assist laboratories in identifying the demand and scope for conducting cytogenetic studies on individual cases.^8^

## METHODOLOGY

This is a retrospective study conducted at the Cytogenetics Laboratory of Indus Hospital and Health Network, Karachi, from January 2020 to May 2025. All requests via consecutive sampling over the defined study period, all, i.e., 348 requested karyotype cases on peripheral blood with suspicion of constitutional chromosomal abnormalities were included.

### Ethical Statement:

### Ethical approval:

It was provided by Institutional Review Board IHHN-IRB NUMBER: IHHN_IRB_023_05_003. As this was a retrospective analysis of laboratory data, exemption was granted from complete IRB review. Patient confidentiality was strictly maintained throughout the study.

### Criteria: 

The following inclusion and exclusion criteria were applied;

- All karyotype cases on peripheral blood with suspicion of constitutional chromosomal abnormalities were included.
- All samples requested for evaluation for neoplastic disorder through karyotyping were excluded.
- Data were extracted from the electronic medical record of the hospital.

The karyotype analysis is done on peripheral blood received in a sodium heparin tube, which is cultured in 10 mL medium with PHA and incubated for 48–72 hours at 37°C (5% CO^2^). Ethidium bromide is added post-culture, followed by 90 minutes of incubation. Colcemid is then introduced to arrest metaphases, with another 30 minutes of incubation. Cells undergo centrifugation and hypotonic wash, then fixed using methanol: glacial acetic acid until a clear pellet forms. Slides are prepared and GTG banding is done, then examined under an inverted microscope. Images of metaphases are captured using Cytovision MB8; at least 20 metaphases are analyzed per patient. Karyotype is described as per ISCN 2020, with analysis performed at 550 bands resolution. The data analysis was done on SPSS 18, frequencies were calculated for clinical indication for advising karyotype, clinical diagnosis, gender, and age distribution, and the most common ultrasound findings.

## RESULTS

**Fig.1:**
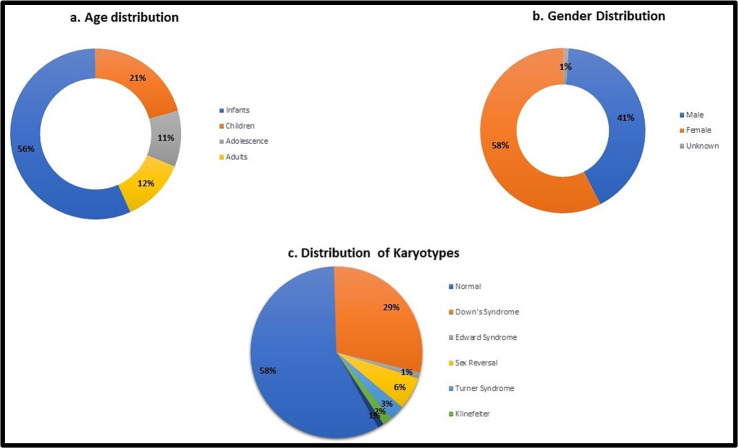
a. Age distribution, b. Gender distribution, c. Distribution of karyotype findings.

**Fig.2:**
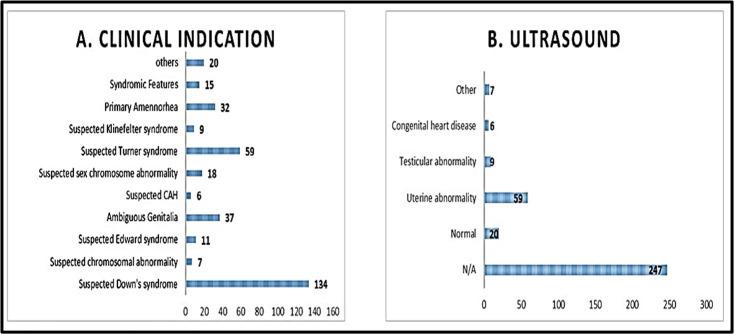
a. Clinical indications for requesting karyotype, b. Ultrasound findings in various disorders.

Total number of cases that were received in the department of cytogenetics for constitutional disorders was 348 out of which 342 were cultured successfully while six had culture failure. There were 201 females and 144 males with a male to female ratio of 0.7 similar ratio of 0.6 was reported by another study from our country,^9^ while three had no gender assignment. Most cases were reported in infancy as shown in Fig.1 with median age being 10 months. Out of the 342 successfully cultured samples 218 patient had normal karyotype which also includes 21 cases of 46,XX in phenotypic male and 46,XY in phenotypic female both. Whereas the frequencies of various abnormalities found are given in Table-II. The most common indication for advising karyotype was suspected Down syndrome (38%), suspected Turner syndrome (11%), and ambiguous genitalia (10%) as shown in Fig.2.

Down Syndrome was suspected in 136 cases and it tested positive 29.2% of the total cases (n=342) and 68.9% of abnormal cases(n=145) and 81% to abnormal karyograms(n=124). Ultrasonographic(U/S) assessment of any kind was done in 25 patients with Down syndrome and out of these six were diagnosed with congenital heart defects which was also the most common U/S findings in those who got the scan done. Turner syndrome contributed to 2.92% of our total cases(n=342) and 6.9% abnormal cases(n=145), Edward contributed to 1.1% of the total cases and 2.76% of abnormal cases(n=145), Klinefelter 1.7% of total cases(n=342) and 4% of abnormal cases(n=145). Three patients got pelvic U/S done and two of the three showed a rudimentary uterus. Out of the 32 patients who came with the complaint of primary amenorrhea, one was diagnosed with Turner Syndrome, one was diagnosed with X iso-Chromosome-X syndrome, two were diagnosed as phenotypic female with male karyotype and one 46,XX,add(15)(q26.3). Total 26 patients with primary amenorrhea were underwent U/S and all patients had abnormal uterine findings with a rudimentary or infantile uterus being the most common finding. Only one patient had normal ovaries rest all had abnormal findings in the ovaries as well.

Out of the 37 patients who came with evaluation of ambiguous genitalia, two had culture failure, 14 were normal, seven were phenotypic male with female karyotype, three were phenotypic female with male karyotype and one had 46,XX,t(5;15)(q22;q26.1) karyotype remaining ten patients had normal karyotype. Out of the total 11 phenotypic female patients, four got U/S done, and all four showed uterine abnormalities. Out of 23 phenotypic males, 10 got U/S done, three were normal rest all showed undescended testis as given in Table-I.

**Table-I:** Individual Disorders and their frequencies in Karyograms

Karyotype	No. of cases	% Of total cases for the disorder	% Contribution to the total successfully cultured 342 samples
Down Syndrome	100		29.2
47,XX+21 Or 47,XY,+21	93	93%	27.2
Robertsonian Translocation	4	4%	1.16
Mosaic Down Syndrome	2	2%	0.58
Down Syndrome with other structural abnormalities	1	1%	0.29
Ambiguous Genitalia (Sex Reversal)	21		6.14
Male 46,XX	11	52.4%	3.21
Female 46,XY	10	47.6%	2.92
Turner syndrome	10		2.92
Homogenous 45,X	6	60%	1.75
i(Xq)	2	20%	0.58
45,X mosaic	2	20%	0.58

**Table-II:** Comparison of relative frequencies of various chromosomal abnormalities to other relevant studies.

Karyotype	Present study		Relative Frequency (%)					
	No.	Relative Frequency (%)	Khan G. et al^9^	Nielson et al.^23^	Hamerton et al.^22^	Aboussair N et al.^18^	Balkan M. et al.^13^	Zohoncon TM et al.^16^
Down Syndrome	100	68.9	46.6	27.6	31.6	70.2	58.3	87.5
Edward Syndrome	4	2.7		3.8	3.1	1.5	0.4	
Sex Reversal	21	14.4	23.7			3.2	2.8	10.3
Turner Syndrome	10	6.9	2.1	13.0	1.6	8.1	12.3	3.44
Klinefelter	6	4.1		36.3	27.6	2.5	12	12.5
Other structural Abnormalities	4	2.7		2.6	27.6		14.2	

## DISCUSSION

Understanding genetic abnormalities elucidates disease mechanisms and advances diagnosis, treatment, and genetic counseling strategies. Out of the 342 successfully cultured cases, 58% were normal as shown in Fig.1. The rate of detection of chromosomal abnormality in our study was 42% in 342 cases, closer to 35.8% reported in another study from Pakistan,^9^ varying frequencies of 29.3%^4^ 28.3%^6^, 28.6%^10^, 17.5%^11^ and 3.8%^12^ have been reported. The broad variation in chromosomal aberration frequencies may stem from differences in patient selection criteria and cytogenetic techniques employed. In our study from 42% of these abnormal karyograms, 71% was contributed by numerical abnormalities, 6.8% by structural abnormalities and 1.3% mosaic. Our study was comparable in terms of numerical and structural abnormalities prevalence with Duarte et al, Santos et al and Balkan et al at 43.7%^4^, 18%^10^ and 20%^13^ for structural anomalies where as 56.3%^4^, 82%^10^ and 80%^13^ for numerical anomalies, Polipalli et al. found a prevalence of 4.86% for structural anomalies and 95.14% for numerical anomalies,^14^ and Shawky et al. reported 78.7% for numerical anomalies and 21.3% for structural anomalies.^15^ A study observed a higher prevalence of structural anomalies 78.37% compared to numerical anomalies 21.62% in contrast to our study.^16^ The patients were predominantly female with a male to female ratio of 0.7, which differed with findings from studies conducted in Africa, Europe, and America.^2^,^4^,^13^ The mean age of consultation in our study was 5.96 years ± 8.92, ranging from one day to 55 years, studies from Turkey, Brazil and Saudi Arabia reported mean ages of 14.3^5^, 11.3^10^ and 14.3^13^ years respectively. Our major patient population is infants and children; other centers may differ demographically.

Down syndrome along with being the most requested for karyotype analysis (38%) was also the most frequent to be diagnosed at 68.9% in all cases diagnosed with an abnormal karyograms. This value is similar to that of another survey which reported a frequency of Down syndrome in patients with abnormal chromosomes to be 87.5%.^16^ The frequency of Down syndrome was found to be 67.9 % in Mokhtar et al.^17^ and by Aboussair N et al.^18^ was 70.2% and in our study 74% of the patients with Down syndrome were diagnosed in their first year of life, with 50% of them being diagnosed within one month of life. Only 22 out of 50 cases which were diagnosed in first month of life, were diagnosed within first week of life because parents may avoid considering Down Syndrome due to limited awareness, cultural stigma, and insufficient emotional, social, and economic support systems. 25% of the patients diagnosed with Down syndrome underwent U/S assessment and 24% of those were diagnosed with congenital heart disease which was also the most common U/S finding in those who got the scan done. Asim A in his study has stated that the incidence of congenital heart disease in patients of Down syndrome is 50%.^19^ And they are by far the most common and leading cause associated with morbidity and mortality in patients with Down syndrome, especially in the first two years of life.^20^,^21^

Approximately one in every 2000 to one in 2500 live female births are affected by Turner syndrome.^22^ Turner syndrome contributed to 2.92% of the total cases and 6.9% abnormal cases in our study, our frequency was similar to those found by Aboussair N et al. at 8.1%, whereas varying frequencies as 3.44%^1^, 12.3%^13^, 1.6%^18^, 27%^21^ and 13%^23^ were also reported. Monosomy X, was found in 60%, mosaic X in 20%, and isochromosome X syndrome in 20% of the cases of Turner syndrome. One case in Zohoncon TM and 6.2% in Aboussair N and 19.6% in Balkan M. Edward contributed to 1.1% of the total cases and 2.7% of abnormal cases, 0.4% in Balkan M. et al, 1.5% in Aboussair N et al., 3.1% and 3.% in Hamerton et al. and Nielson et al. Klinefelter 1.7% or total cases and 4% of abnormal cases, our frequencies differed from the studies which reported much higher frequencies at 12%^13^, 12.5%^18^, 27.6%^22^ and 36.6%.^23^ Three patients got pelvic U/S done and two of the three showed rudimentary uterus.

Out of the 32 patients who came with the complaint of primary amenorrhea five had abnormal karyotype, of these three were diagnosed with sex reversal 60%, one with monosomy X Turner Syndrome 20%, one was diagnosed with X iso-chromosome-X syndrome 20%, similar findings were seen by Pal AK et al. with sex reversal being culprit in 43% of primary amenorrhea cases. Monosomy X was the second most common abnormality found in patients with primary amenorrhea was, i.e., pure Turner syndrome (45,X) in 26% cases, isochromosome iXq seen in 4.3% of the cases and others contributed 26.7%^24^ 26 patients underwent U/S and all patients had abnormal uterine findings with rudimentary or infantile uterus being the most common finding. Only one patient had normal ovaries rest all had abnormal findings in ovaries as well.

Out of the 37 patients who came with evaluation of ambiguous genitalia, two had culture failure, 14 were normal, seven were phenotypic male with karyotype of female, three were phenotypic female with karyotype of male and one had 46,XX,t(5;15)(q22;q26.1) karyotype remaining ten patients had normal karyotype. 11 phenotypic female patients four got U/S done and all four showed uterine abnormalities. Out of 23 phenotypic males, 10 got scans done, three were normal rest all showed undescended testis.

### Strengths of the study:

The present study holds several notable strengths. It is the first of its kind to report the prevalence and pattern of constitutional chromosomal abnormalities in the province of Sindh, thereby providing valuable baseline cytogenetic data for this region. The inclusion of a diverse patient population, encompassing various ethnic and socioeconomic backgrounds, enhances the generalizability of the findings. Furthermore, the study’s correlation of cytogenetic results with clinical parameters adds important clinical relevance, helping to better understand the phenotypic implications of chromosomal disorders. By contributing to the limited national database on chromosomal abnormalities in Pakistan, this research fills a crucial knowledge gap and serves as a foundation for future studies and policy development. The use of standardized cytogenetic techniques further strengthens the reliability and accuracy of the findings.

### Limitations of the study:

The study also has certain limitations that must be acknowledged. The absence of specific clinical details such as maternal and paternal ages, obstetric history, and family history limited the ability to explore potential associations, particularly between maternal age and the incidence of Trisomy 21. Being a single-center study, the findings may not fully represent the distribution of chromosomal abnormalities across the wider population. In addition, the sample size may not have been large enough to determine precise prevalence rates for rarer chromosomal anomalies. The lack of molecular confirmation methods, such as FISH or array-CGH, restricted the detection of submicroscopic chromosomal abnormalities.

## CONCLUSION

This study has highlighted a significant prevalence of chromosomal abnormalities among the Pakistani population. Numerical abnormalities were observed more commonly than structural abnormalities, and autosomal chromosomes were more frequently affected than sex chromosomes. Our findings offer key insights to guide essential research and advance genetic healthcare initiatives in Pakistan.

### Recommendations:

There are insufficient data in the current epidemiology of genetic disorders in Pakistan. The efficient genetic registries, genetic databases, and continuous investments in genetic research are key to successful public health interventions. A standard protocol needs to be designed at the government level for history taking and registry of patients with suspected constitutional chromosomal abnormality in order to generate the constitutional chromosomal abnormalities’ genetic landscape in Pakistan. For a better frequency estimation in the Pakistani population, a diverse group needs to be studied, including centers that deal with a larger population.
